# Incidence, knowledge, attitude and practice toward needle stick injury among nursing students in Saudi Arabia

**DOI:** 10.3389/fpubh.2023.1160680

**Published:** 2023-05-04

**Authors:** Khalid Al-Mugheed, Sally Mohammed Farghaly, Nadiah A. Baghdadi, Islam Oweidat, Majdi M. Alzoubi

**Affiliations:** ^1^Adult Health Nursing and Critical Care, Riyadh Elm University, Riyadh, Saudi Arabia; ^2^Department of Nursing Management and Education, Princess Nourah Bint Abdulrahman University, Riyadh, Saudi Arabia; ^3^Department of Nursing Management, Zarqa University, Zarqa, Jordan; ^4^Community Health Nursing, Al-Zaytoonah University of Jordan, Amman, Jordan

**Keywords:** needle stick injury, nursing students, incidence, knowledge, attitude, practice

## Abstract

**Background:**

Needle stick injuries constitute the greatest threat to nursing students during clinical practice because of accidental exposure to body fluids and infected blood. The purpose of this study was to (1) determine the prevalence of needle stick injuries and (2) measure the level of knowledge, attitude and practice among nursing students about needle stick injuries.

**Methods:**

Three hundred participants undergraduate nursing students at a private college in Saudi Arabia were included, of whom 281 participated, for an effective response rate of 82%.

**Results:**

The participants showed good knowledge scores with a mean score of 6.4 (SD = 1.4), and results showed that students had positive attitudes (Mean = 27.1, SD = 4.12). Students reported a low level of needle stick practice (Mean = 14.1, SD = 2.0). The total prevalence of needle stick injuries in the sample was 14.1%. The majority, 65.1%, reported one incidence in the last year, while (24.4%) 15 students reported two incident of needle stick injuries. Recapping was the most prevalent (74.1%), followed by during injection (22.3%). Most students did not write a report (77.4%), and being worried and afraid were the main reasons for non-reports (91.2%). The results showed that female students and seniors scored higher level in all needle stick injuries domains (knowledge, attitude and practice) than male students and juniors. Students who had needle stick injuries more than three times last year reported a lower level of all needle stick injury domains than other groups (Mean = 1.5, SD =1.1; Mean = 19.5, SD =1.1; Mean = 9.5, SD =1.1, respectively).

**Conclusion:**

Although the student’s showed good knowledge and positive attitudes in NSI, the students reported a low level of needle stick practice. Raising awareness among nursing students and conducting continuing education related to sharp devices and safety and how to write an incident reporting is highly recommended.

## Background

Nearly all nursing students experience some adverse effects or challenges in their clinical or training placement that compromise their safety or the patient’s safety (1). One primary challenge nursing students face is needle stick injuries (2). Needle Stick Injury (NSI) is a nonintentional wound or injury that results from needles connected with Intravenous (IV) and blood transfusion sets (3). Exposure to contaminated needles may expose those injured to the potential risk of pathogens such as Hepatitis B (HBV), Hepatitis C (HCV) and Human Immunodeficiency Virus (HIV), with a post-exposure transmission rate of 30%, 5–10, and 0.4%, respectively, (4).

Percutaneous exposure to body fluids and blood during sharp equipment and needle stick infection is considered the main occupational hazard for mortality and morbidity risk of pathogens in the clinical environment (5). The risk of exposure and transmission to blood-borne pathogen infections through needle sticks and sharp injuries is very high among healthcare students, specifically nursing students (6–8). The prevalence of NSI among nursing students varied in numerous studies worldwide, ranging from 11.8 to 85.0% (6, 9). In recent systematic review showed prevalence of NSI in developing countries was significantly higher than in developed countries among nurses (10). Alsabaani et al. found that the prevalence of needle stick injury among healthcare workers in Saudi Arabia was 11.57% (11). In a recent study conducted among healthcare workers in Saudi Arabia, the prevalence of needle stick injury was 22.2% (12).

Most NSI incidents occurred through drug preparation, administration, recapping, holding syringes without a suitable container, opening needle caps, suturing and blood sampling (12–15). Other causes include inadequate staff, lack of training, lack of experience with infection-control standards, and insufficient appropriate resources (16, 17).

Several psychological consequences of NSI have been noted. For example, a systematic review found that depression, fear, and anxiety were the main psychological effects of NSI among nursing students, and the study suggested that students in their clinical fields need more support and counseling services after being exposed to injury (18). Another study in a psychiatric trauma clinic reported that exposure to NSI can create mental problems (19). Thus, policymakers and teachers need to direct their attention to the mental and physical consequences of NSI.

The National Institute for Occupational Safety and Health has issued prevention guidelines for healthcare providers and students to control needle stick injuries (20). Post-exposure prophylaxis plays a significant role in preventing a person from HIV, HBV and HCV and their chronicity (21). Therefore, promptly reporting an incident is crucial for a student or healthcare personnel; failure to do that may result in catastrophic consequences (19). Using safer devices, administering Hepatitis B vaccination, pre-exposure prophylaxis and administering post-exposure prophylaxis contribute to decreasing the incidences drastically and are cost-effective (20).

Most studies conducted in Saudi Arabia included healthcare workers and ignored the nursing students. In fact, the nursing students are more vulnerable to NSIs because of their lack of clinical experience. The current study aimed to address this issue by (1) assessing the prevalence of NSI and (2) measuring the level of knowledge, attitude and practice among nursing students about NSI.

## Methodology

### Study design

This cross-sectional and descriptive study was conducted among undergraduate nursing students.

### Sample size and study setting

The study was performed in a private college in Saudi Arabia. This college has seven branches in Saudi Arabia (Riyadh, Jeddah, Najran, Abha, Tabuk, Al Madinah Al Munawwarah and Dammam) and includes more than 440 undergraduate nursing students. G* power software was used to calculate the sample size. Based on an estimated effect size of (d) = 0.7, *ά* = 0.05, power = 0.95, the required sample size was estimated at 254 to run a paired sample t-test. Three hundred students were asked to participate, of whom 281 participated, for a response rate of 82%. In literature reviews, as a general rule, a sample size of 200–300 is considered sufficient for this type of study (22, 23). This study specifically focuses on students from the second to the final year, which is their clinical practice time, where they are at high risk of contracting NSI through a particular procedure–for instance, applying intravenous cannula and venipunctures, among others.

### Data collection tool

The researchers prepared the questionnaire based on NSI studies (6, 8). The questionnaire had four parts. The first part consisted of two parts, the first part asked for demographic data such as gender, age, year of study, previous education of NSI, the second part were related Prevalence of NSI such as number of NSI incidences last year, how the incidences occurred, writing an incident report and reasons for not reporting. Part two comprised eight knowledge questions related to general information about NSI, such as definitions of NSI, common causes, sharps containers, and knowledge about blood-borne diseases. Each question included two options (True, False). The true answer was scored 1, while the false answer was 0. The total knowledge score could range from 0 to 8, where a higher score represents a higher knowledge level. The third part comprised seven statements related to attitudes toward NSI perceptions. Responses were from 1 to 5, using a 5-point Likert-type scale (“Strongly Agree 5,” to “Strongly Disagree 1”). The positive score ranged from 5 to 35, where a higher score represents a higher knowledge level. The last part contained statements about five practice-related needle stick injuries, including standard precautions and vaccination status for the protections, preventions precautions about NSI and what you should do after an incidence occurs Post-exposure prophylaxis (PEP). These statements used a 5-point Likert-type scale (always 5, often 4, sometimes 3, rarely 4, never 5), with a total score ranging from 5 to 25, where a higher score represents a higher practice level.

Three doctors of nursing and infection control reviewed the content validity and item language to evaluate whether the questionnaire items effectively captured the most information students need to prevent NSI. The review committee recommended that the questionnaires cover most NSI prevention information, the language being easy to understand and straightforward. A pilot was conducted with information from ten respondents. The questionnaires had good reliability and internal consistency with Cronbach’s alpha of 0.80, 0.71, and 0.72 in terms of knowledge, attitude and practice categories, respectively. A good degree of inter-rater inter-observer reliability was found between questionnaire items. The average interclass correlation coefficient was 0·81 with a 95% confidence interval from 0·79 to 0·86 [*F* = 4·5, *p* = 0·01].

### Data collection

After the ethical approval was secured from the ethical review committee at the university where the study was held, with reference number (2022/77/10), the researchers recruited students through an online survey widely used for surveys worldwide. An online survey in Google forms was sent to students *via* social media such as Facebook and WhatsApp. Information, including the study’s aim and link attachment, was sent along with instruments to guide the participants in filling out the survey. Agreement to participate in the study was obtained when the participants completed the online survey. Reminders were sent frequently to remind students to fill out the survey. The study was open from January until December 2022. The study was open from January until December 2022. To prevent the duplicate submission of the survey, participants could only fill out the survey once.

### Statistical analysis

The data were imported from the online survey into the Statistical Package for the Social Sciences (SPSS) Version 20. Descriptive statistics percentage, frequency, mean, and standard deviation were calculated. Independent t-tests and a One-Way Analysis of Variance (ANOVA) were applied to compare the means of total knowledge with sample characteristics. A value of *p* of <0.05 was considered significant.

## Results

A total of 281 students completed the electronic survey, which gave an overall 81.5% response rate. The mean age was 24.9 (SD = 1.3) years. Most participants were female students 65.3% (*n* = 176). Of the total sample, 46.1% (*n* = 108) were in their fifth year of study. Of the 281 students, (*n* = 68) reported having experienced needle stick injuries. Regarding previous NSI education, 59.5% of them had previous education. The total prevalence of NSI among our sample was 14.1%. The majority of students *n* = 47 (65.1%) reported one incidence in the last year, while *n* = 15 (24.4%) students reported two incidences. Recapping incidents occurred the most (74.1%) which is considered as a wrong practice, followed by during injection (22.3%). Most students did not write a report (77.4%) and being worried and afraid were the main reasons for non-reports (91.2%; Table 1).

**Table 1:** Sample characteristics.

A. Sample characteristics	*N*	%
Gender		
Male	105	34.7
Female	176	65.3
Age		
18–22	128	47.8
23–27	85	27.5
28	68	21.7
Study year		
Second year	25	5.5
Third year	51	20.5
Fourth year	97	27.9
Fifth year	108	46.1
Previous education about needle stick injuries (NSI)		
Yes	175	59.5
No	106	40.5
B. Prevalence of NSI		
No. of NSI incidence last year (*N* = 68)		
Once	47	65.1
Twice	15	24.4
More than two	6	10.5
How incidence occurred (*N* = 68)		
During injection	13	22.3
While recapping	48	74.1
Wound suturing	4	2.2
Lumber puncture	3	1.4
NSI Incidence report (*N* = 68)		
Inform clinical instructors	3	2.5
Write a report	16	20.1
Did not write a report	49	77.4
Reasons for not reporting (*N* = 49)		
Did not know the standard	11	7.2
Neglected	4	1.6
Worried and afraid	34	91.2

Total NSI knowledge scores for the participating students ranged from 1 to 8, with a mean score of 6.4 (SD = 1.4). The range of correct answers to each question ranged from 76.2 to 92.2%, which was 77.0% of the highest possible score. “Safer devices and technics and gloves are needed to avoid needle stick accidents” received the highest correct answer percentage (92.2%). The lowest correct answers percentage (75%) was for the item “is the maximum capacity for a sharps container” (76.2%; Table 2).

**Table 2:** Knowledge of needle stick injury.

	Statement	Options		%
1	Needle stick injury is defined as wounds caused by needles that accidentally puncture the skin.	T	240	89.1
		F	41	10.9
2	Recap of the syringe after performing nursing interventions is recommended to decrease the risk of needle stick injury (False*)	T	49	12.8
		F	232	87.2
3	Hepatitis B can be prevented by vaccine?	T	211	81.5
		F	70	18.5
4	Safer devices and technics and gloves are needed to avoid needle stick accidents?	T	258	92.2
		F	23	7.8
5	Hepatitis B & C, HIV, are the blood-borne pathogens that health care providers are most commonly exposed to when they experience NSI?	T	225	82.1
		F	56	17.9
6	75%, is the maximum capacity for a sharps container?	T	199	76.2
		F	82	23.8
7	Wash area with soap and water is recommended to decrease the risk of infection immediately after experiencing NSI	T	205	78.8
		F	76	21.2
8	Dispose in a sharps container practice is recommended to decrease the risk of injury?	T	222	81.8
		F	59	18.2

Table 3 shows the mean for the attitude of needle stick injury among nursing students. In general, results showed that students had positive attitudes (Mean 27.1, SD = 4.12). For example, two-thirds of students had taken a hepatitis B vaccination. Likewise, 60.2% reported that they worried about NSIs, and 72.4% believed that an NSI was preventable. Only half of the students were more concerned about patient care. Most students perceived NSI as the most common event (79.8%). Finally, 60.4% of students agreed that NSI was neglected.

**Table 3:** Attitude of needle stick injury.

	Attitude items	Strongly agree		Agree		Neutral		Disagree		Strongly disagree	
		*N*	%	*N*	%	*N*	%	*N*	%	*N*	%
1	Hepatitis B vaccination have taken	175	70.3	50	15.1	20	5.7	15	3.2	21	5.7
2	Worried about NSI	45	14.5	158	60.2	36	11.7	23	7.5	19	6.1
3	NSI is preventable	181	72.4	55	13.8	10	1.6	20	6.4	15	5.8
4	More concerned on patient care	87	25.5	140	50.1	15	8.8	22	9.4	17	6.2
5	NSI is most common event	201	79.8	54	10.3	10	3.8	10	3.8	6	2.3
6	Report NSI immediately	205	80.2	66	16.8	10	3.0	0	0	0	0
7	NSI is neglected	88	28.6	159	60.4	15	4.1	10	3.8	9	3.1

Students reported a low level of needle stick practices (*M* = 14.1, SD = 2.0). Half of the students always recapped needles before discarding them (50.1%). Approximately one-third of the students reported wearing gloves before venipuncture/injections (35.4%). Only one-quarter of the students reported using one-handed recapping, using PPE during procedures, and rinsing with soap and water after NSI (23.6, 22.8, 20.6%), respectively (Table 4).

**Table 4:** Practice of needle stick injury.

	Statement	Always		Often		Sometimes		Rarely		Never	
		*N*	%	*N*	%	*N*	%	*N*	%	*N*	%
1	Recap needles before discarding	150	49.1	65	25.7	44	16.5	15	6.7	7	2.0
2	Wear gloves before venipuncture/injections	100	35.4	135	46.5	20	7.4	10	4.6	16	6.1
3	One hand method of recapping done	67	23.6	48	17.4	64	23.1	17	5.7	85	30.2
4	Use PPE during procedures	61	22.8	45	18.4	33	10.4	54	19.8	88	28.6
5	Rinse with soap and water after NSI	55	20.6	28	7.3	35	11.6	52	20.4	111	40.1

The total knowledge, attitude and practice scores were compared among nursing students. The results showed that female students and seniors scored higher in all NSI domains than male students and juniors. Students who reported NSI incidences more than three times in the last year had a lower level of all NSI domains than other groups (*M* = 1.5, SD =1.1; *M* = 19.5, SD =1.1; *M* = 9.5, SD =1.1, respectively; Table 5).

**Table 5:** Comparisons of needles stick injuries mean score and selected demographics.

	Knowledge *M* (SD)	*t*	*P*	Attitude M (SD)	*t*	*P*	Practices M (SD)	*t*	P ***
Gender *									
Male	4.1 ± 1.1	5.20	0.94	20.3 ± 2.1	4.22	0.81	10.3 ± 1.4	3.20	0.84
Female	5.2 ± 1.3			21.6 ± 2.8			11.6 ± 2.8		
Previous education about NSI *									
Yes	5.7 ± 2.3	1.87	0.01	22.3 ± 2.2	1.8	0.03	11.8 ± 2.4	2.6	0.03
No	2.8 ± 1.5			19.2 ± 1.8			10.5 ± 1.1		
Year of Study **									
Second year	2.2 ± 1.4	1.20	0.01	22.8 ± 1.5	2.30	0.02	10.9 ± 1.8	1.96	0.02
Third year	3.1 ± 2.1			20.5 ± 1.7			11.8 ± 2.2		
Fourth year	3.6 ± 1.5			25.5 ± 1.4			12.2 ± 1.2		
Fifth year	4.7 ± 1.1			26.3 ± 1.2			14.5 ± 1.8		
No. of NSI Incidence last year**									
Once	3.8 ± 1.9	1.5	0.13	21.3 ± 1.9	2.9	0.22	10.3 ± 1.7	1.8	0.18
Twice	2.1 ± 1.7			19.8 ± 1.3			9.8 ± 1.2		
More than Three	1.5 ± 1.1			19.5 ± 1.1			9.5 ± 1.1		
How incidence occurred **									
During Injection	2.5 ± 1.9	1.66	0.15	22.1 ± 2.6	1.87	0.11	12.1 ± 1.3	3.0	0.03
While Recapping	3.1 ± 2.1			19.2 ± 1.3			9.7 ± 1.2		
Wound Suturing	2.3 ± 1.9			21.5 ± 1.9			10.5 ± 1.7		
Lumber Puncture	1.6 ± 1.1			20.4 ± 1.7			10.9 ± 1.8		

*Independent *t*-tests; **ANOVA; ***Significant at *p* ≤ 0.05.

## Discussion

Needle stick injuries constitute the greatest threat to nursing students during clinical practice because of accidental exposure to body fluids and infected blood affect the patient safety (24–26). The current study demonstrated significant results related to nursing students in terms of knowledge, attitudes, and practices related to needlestick injuries. Notably, the students had adequate knowledge of NSI. This result aligns with a study conducted in Northern Cyprus, where participants showed inadequate knowledge (27) and contrasted with international studies that showed the students had inadequate knowledge of NSI (8, 28, 29). Such a high score indicates that nursing students in the current study had sufficient knowledge regarding NSI, confirming that the nursing school offered special courses such as infection control to students before starting clinical practice (30). More than half of the nursing students were senior students and had previous education about NSI, meaning they have high exposure to the causes and risk factors of needle stick injuries.

Participants’ attitudes toward NSI were positive and dissimilar to a previous study (31) but similar to a national study that showed positive attitudes toward NSI among healthcare providers (21). Students’ positive attitudes confirm that nursing schools are the proper place to raise students’ awareness and behavior in terms of NSI attitudes before transmission in clinical practice and enhance their decision-making skills (32).

Regarding post-exposure treatment for NSI and methods of prevention practice, the mean was low. For example, less than half of the participants reported consistently engaging in post-exposure treatment for NSI, and this finding is consistent with previous studies (8, 28). Inadequate post-exposure treatment for NSI practice has been considered the most significant risk of NSI, leading to unsafe practice during clinical practice (33, 34). However, preventive and post-exposure measures topics should be taught and mandatory in all nursing schools’ curriculums before starting clinical practice (35).

The total prevalence of NSI among our sample was 14.1%. These percentages are lower than different studies, such as Germany (21.4%) (36) and Taiwan (18.2%) (37). A recent systematic review and meta-analysis showed that the prevalence of NSI among nursing students in Asia countries was higher than in Europe (39.7%) (38). Failure to recap the needles was reported as a common cause of NSIs, similar to several studies (6, 8). Finally, many students did not fill out an incident report because they were worried and feared being blamed. Under-reporting NSI is a major clinical challenge that may have undermined the validity of existing data regarding this issue (38, 39).

In the current study, the results showed no significant differences in NSI domain scores between the participants regarding genders, although females demonstrated higher scores on all domains than male students. These results were similar to study nursing students in Turkey, where students showed a different level of NSI knowledge (40). Conversely, Jordanian nursing students did not show significant differences in knowledge of NSI (8). The varied level of knowledge among the students may be related to infection control courses and contents in the different nursing schools.

Our study shows that senior nursing students had better scores than junior students, similar to (8). These results can be because senior nursing students had more experience in infection control practices and had taken more courses. This result emphasized that infection control courses would be better given a preparatory year to ensure that students had adequate knowledge before shifting to clinical practice.

In this study, nursing students with more than three incidences in the last year of NSI showed lower overall NSI knowledge domains than other groups. This result may be explained because students were inexperienced or stressed while carrying out invasive procedures. Underdeveloped skills and lack of clinical experience may be associated with an increased risk of NSIs among health professional students (41–43). However, the risk of NSIs is linked with clinical skill and may also be associated with the frequency of procedures and inherent hazards and influences individual health management (44, 45).

This study had limitations. Using online surveys in data collection could lead to inaccurate results and recall bias. Because the study was conducted in one private university and one nursing faculty with different branch, this may reduce the generalizability of our findings. Nonetheless, this study helps fill a knowledge gap because there are few studies in nursing education in Saudi Arabia about injuries and safety issues. Future studies, including students in healthcare professions, are highly recommended.

## Conclusion

Although the student’s showed good knowledge and positive attitudes in NSI, the students reported a low level of needle stick practice. The exposure of nursing students to needle stick injury and its non-reporting remains a persistent challenge. Raising awareness among nursing students and conducting continuing education related to sharps devices and safety is highly recommended. Policymakers should implement several initiatives to reduce NSI incidences, such as safe injection practices, safety precautions, reporting systems, and the use of post-exposure prophylaxis.

## Data availability statement

The raw data supporting the conclusions of this article will be made available by the authors, without undue reservation.

## Ethics statement

The studies involving human participants were reviewed and approved by After the ethical approval was secured from the ethical review committee, with reference number (2022/77/10). The patients/participants provided their written informed consent to participate in this study.

## Author contributions

All authors listed have made a substantial, direct, and intellectual contribution to the work and approved it for publication.

## Funding

This research was funded by Princess Nourah bint Abdulrahman University Researchers Supporting Project number (PNURSP2023R293), Princess Nourah bint Abdulrahman University, Riyadh, Saudi Arabia.

## Conflict of interest

The authors declare that the research was conducted in the absence of any commercial or financial relationships that could be construed as a potential conflict of interest.

## Publisher’s note

All claims expressed in this article are solely those of the authors and do not necessarily represent those of their affiliated organizations, or those of the publisher, the editors and the reviewers. Any product that may be evaluated in this article, or claim that may be made by its manufacturer, is not guaranteed or endorsed by the publisher.
